# TSLP impairs mucociliary transport by inhibiting JAK signaling in both central and peripheral airways

**DOI:** 10.1007/s00424-026-03201-z

**Published:** 2026-08-04

**Authors:** Hatsumi Sugiyama, Tsutomu Tamada, Koji Murakami, Hidemi Aritake, Risako Shionoya, Mitsuhiro Yamada, Masayuki Nara, Itsuro Kazama, Takashi Nakahari, Hisatoshi Sugiura

**Affiliations:** 1 Department of Respiratory Medicine, Tohoku University Graduate School of Medicine, 1-1 Seiryo-machi, Aoba-ku, Sendai, 980-8574 Japan; 2 Division of Respiratory Medicine, Tohoku Medical and Pharmaceutical University, 1-15-1 Fukumuro Miyagino-ku, Sendai, 983-8536 Japan; 3 National Hospital Organization Akita National Hospital, Yurihonjo, Japan; 4 Miyagi University School of Nursing Graduate School of Nursing, Kurokawa-gun, Japan; 5 Research Unit for Epithelial Physiology, Research Organization of Science and Technology, BKC, Ritsumeikan University, Kusatsu, Japan; 6 Medical Research Institute, Kyoto Industrial Health Association, Kyoto, Japan

**Keywords:** MCT, Cilia, TSLP, JAK, Bronchial asthma

## Abstract

Mucociliary transport (MCT) is the dominant mechanical host defense system in human airways. Although the importance of the peripheral airway in the pathophysiology of bronchial asthma has recently attracted attention, the characteristics of MCT in the peripheral airway during asthma remain unclear. This study aimed to investigate MCT velocity in the central and peripheral airways and the effects of thymic stromal lymphopoietin (TSLP) on MCT. Central and peripheral airways were isolated from freshly obtained porcine airways immediately after slaughter. The airway specimens were mounted in a custom-built fluorescence microscopy–based measurement system. MCT velocity and ciliary beat frequency (CBF) were measured under the following conditions: without stimulation, in the presence of a low concentration of acetylcholine (ACh), and after stimulation TSLP, which reflects the pathophysiological condition of bronchial asthma. MCT velocity in the peripheral airways was slower than that in the central airways at less than one tenth (median: 75.40 μm/sec vs. 2.63 μm/sec). Under physiologically low ACh concentrations, MCT velocity was increased in the peripheral airway but not in the central airway. TSLP addition partially inhibited MCT velocity in both the central and peripheral airways by approximately two-thirds to one-half. TSLP also partially inhibited the CBF in both the central and peripheral airways from approximately two-thirds to one-third. Furthermore, TSLP receptor (TSLPR) expression was confirmed in airway tissues by immunohistochemical staining. The inhibitory effects of TSLP on MCT velocity were completely abolished by pre-incubation with a Janus kinase (JAK) inhibitor. In conclusion, TSLP has the potential to reduce MCT velocity, especially in the peripheral airways, by inhibiting TSLPR signaling pathways, resulting in impaired CBF. The characteristics of MCT differ between the central and peripheral airways. Importantly, TSLP-induced MCT impairment may aggravate mucus plug formation in the peripheral airways in patients with bronchial asthma.

## Introduction

The human lung is constantly exposed to foreign particles inhaled during respiration, and mucociliary transport (MCT) is the primary mechanical host defense system in the human airways [1–3]. MCT is required to protect both the large central and small peripheral airways against inhaled microorganisms and other particulates [4]. Although the central and peripheral airways differ in structure and function, studies investigating the characteristics of MCT in these regions are limited. MCT involves the airway surface liquid (ASL) and ciliary beating. In the central airways, toll-like receptor (TLR) signaling and oxidative stress induce changes in the volume and pH of ASL, as well as alterations in ciliary beat frequency (CBF) [5–8]. The peripheral airways differ anatomically and physiologically from the central airways. Compared to the central airways, the peripheral airways lack cartilage and submucosal glands; contain fewer elastic fibers [9]; exhibit slower airway clearance [10, 11]; and have higher cystic fibrosis transmembrane conductance regulator activity, a more alkaline ASL [12], and shorter cilia [10, 13]. However, the differences in MCT between the central and peripheral airways and the mechanisms regulating these differences remain unclear.

Severe asthma, characterized by recurrent exacerbations despite high-dose treatment with inhaled corticosteroids and additional controllers, accounts for 5–10% of all asthma cases. In Japan, approximately 1,000 patients with severe asthma die annually [14, 15]. The peripheral airways have been recently found to be a predominant site of airflow limitation, inflammation, and airway hyperresponsiveness in asthma [16, 17]. Mucus plug formation, which exacerbates severe asthma, mainly originates in the peripheral airways and progresses toward the central airways. During this process, the mucosal defense systems across the entire airway are disrupted, resulting in decreased MCT, reduced antimicrobial protein activity, and increased mucin viscosity [18]. MCT impairment also contributes to mucus plug formation [19].

Thymic stromal lymphopoietin (TSLP), a key cytokine involved in type 2 inflammation in asthma, has been implicated in mucus plug formation. Higher TSLP levels are associated with a greater number of mucus plugs, and mice lacking the TSLP receptor (TSLPR) exhibit lower mucus scores [20–23]. A randomized controlled trial also showed that treatment with tezepelumab, a monoclonal antibody that prevented TSLP from interacting with its heterodimeric receptor, was associated with a reduction in occlusive mucus plugs in patients with moderate-to-severe uncontrolled asthma [24]. However, the effects of TSLP on MCT and ciliary motility remain unclear. Thus, this study aimed to evaluate the MCT in the central and peripheral airways and investigate the effects of TSLP on MCT and ciliary beating in both regions. We hypothesized that TSLP exerted an inhibitory effect on MCT.

## Materials and methods

### Separation of central and small airways

Landrace×Yorkshire×Duroc (LYD) crossbred pigs aged 6–8 months were used, with the proportion of female and male swines being almost uniform. Since the samples were collected with the trachea and lungs removed, the sex of the individuals used in this study could not be determined. This process followed the same procedure as in our previous experiment [8]. Fresh swine tracheas and attached lung were obtained from a local slaughterhouse, stored in ice-cold extracellular solution, and transported to our laboratory. Adipose and connective tissues adhering to the outer tracheal surface were removed, and the trachea was cut into 3–4 cm segments as the central airways. From the lungs attached to the trachea, a sonde was inserted into the central side of the bronchus to confirm continuity with the trachea, and the distal bronchi were carefully dissected. Bronchi with an internal diameter < 2 mm and lacking cartilage were defined as the peripheral airways. Clinical trial number: not applicable.

### MCT measurement

**Fig. 1:**
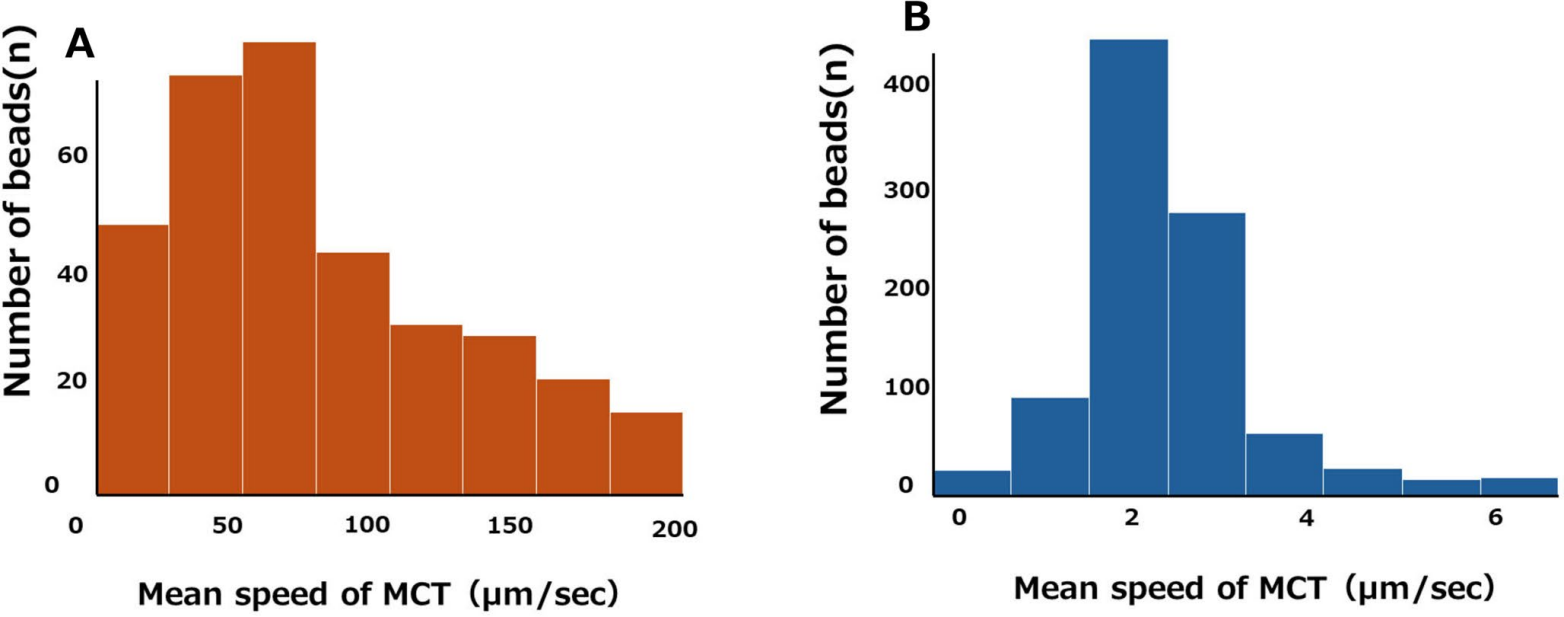
MCT velocity in both central and peripheral airways (*n*=5). MCT velocity is measured by tracking the movement of 1-μm fluorescent beads using a high-speed camera attached to a fluorescence microscope. The histograms show the distribution of MCT velocity measured under identical experimental conditions in the central and peripheral airways. MCT velocity in the central airways is several tens of times higher than that in the peripheral airways

Isolated central and peripheral airway tissue samples were incubated at 4 °C for 24 h in Roswell Park Memorial Institute (RPMI) medium containing 15 ng/mL TSLP. Control samples were incubated in RPMI medium containing an equal amount of phosphate-buffered saline (PBS) instead of the TSLP solution. For Janus kinase (JAK) inhibition experiments, 2.5 µM ruxolitinib (RUXO) or an equivalent volume of dimethyl sulfoxide was added to the TSLP-containing medium under the same conditions. After carefully removing the cartilage and smooth muscle layers, the tracheal tissues were fixed horizontally with the luminal side facing up. The peripheral airway samples were carefully separated from the surrounding pulmonary tissue, opened longitudinally, and mounted similarly (Fig. 1). The mounted airway samples were soaked in standard extracellular fluid (120 mM NaCl, 4.7 mM KCl, 1.13 mM MgCl₂, 1.2 mM CaCl₂, 10 mM glucose, and 10 mM HEPES). To analyze mucociliary transport, fluorescent beads (a 25,000-fold dilution of ECF, 1 μm FluoSpheres Polystyrene Microspheres, YG Microspheres) were added to the apical side of swine tracheal epithelia, and observed using a fluorescence microscope (SZX16, OLYMPUS, Tokyo, Japan). The movement of 1 μm fluorescent beads was recorded at 21–23 °C up to 30 min post bead addition for 2 min every 2 s under fluorescence stereomicroscopy. The microbead movement trajectories and their velocities were automatically quantified using the image processing package Fiji, v1.54f, ImageJ as previously reported [25, 26]. In short, each bead was tracked and analyzed using the TrackMate plugin for Fiji (https://fiji.sc/). Mucociliary transport of the fluorescent beads was quantified using ‘Speed’ indicator, that is the velocity of the flow of fluorescent beads (µm/s). Outliers and beads that stopped moving during acquisition were excluded from the analysis. Among hundreds of beads, the proportion of beads that stopped during the experiments and beads exhibiting only short displacement distances varied among individual animals, but was approximately 6% of the total dataset. Additionally, beads exhibiting velocities too greater than other all beads were also excluded from the analysis, as their movement was considered to be influenced by the perfusate flow. From the histogram shown in Fig. 1, values approximately five times the third-quarter value were clearly determined to be automatically measured trajectories unrelated to the actual movement of the beads and were excluded as outliers. Specifically, outliers were defined as values exceeding 20 μm/s in the peripheral airways and 500 μm/s in the central airways. Only approximately 5% of the beads were excluded on the basis of being identified as outliers. The effect of acetylcholine (ACh) on MCT, was evaluated with measurements performed in the presence and absence of 30 nM ACh. The numbers of pigs used in each experiment are indicted in figure legend. Based on previous reports [5–8], it has been confirmed that airway cells remain active for at least two days after collection. In this study, the MCT experiment was performed only on the day of collection, and it was assumed that the mucus transport capacity of epithelial cells was similarly active. However, samples in which bead movement was not observed during the experiment were considered inactive trachea and were excluded from subsequent analysis (*n* = 2).

### CBF measurement

The central and peripheral airway samples were incubated in RPMI medium containing 15 ng/mL TSLP at 4 °C for 24 h, and RPMI media supplemented with an equal amount of PBS served as controls. The central airway samples were longitudinally incised at the cartilaginous region, and the epithelium and submucosa were manually detached to prepare an epithelial sheet. The peripheral airway samples were trimmed to remove the adjacent tissues and opened longitudinally. Epithelial sheets from central and peripheral airway segments were washed with 500 mM DL-DTT solution and then rinsed thrice with calcium-free extracellular fluid (ECF). The samples were subsequently incubated in protease solution at 37 °C for 30 min, calcium-containing ECF was added, and the mixture was vigorously agitated to dissociate the epithelial cells. Tissue fragments were removed, and the resulting cell suspensions were collected. The cells were set in a micro perfusion chamber mounted on an inverted light microscope (ECLIPSE Ti, Nikon, Tokyo, Japan) connected to a high-speed camera (Photron Ltd., Tokyo, Japan) and video images were recorded for 2 s at 500 fps (frame per second) [27–29]. The stage of the microscope was heated to 37 °C. After the experiments, the CBF was measured using an image analysis program (DippMotion 2D, Ditect, Tokyo, Japan). The method for measuring CBF was performed as previously described in detail [27–29]. Ciliated cells were randomly selected.

### Immunohistochemistry

Tracheal and lung tissues were collected from freshly slaughtered pigs, and 3 mm × 10 mm tissue blocks were excised. Mediastinal lymph nodes were used as positive controls. The samples were fixed in 10% formalin and embedded in paraffin wax. After deparaffinization, non-specific binding sites were blocked by incubation with normal goat serum for 30 min at 21–23℃. The sections were then incubated with rabbit anti-human CRLF2 antibody (1:400 dilution, Sigma-Aldrich) at 4 °C overnight. Subsequently, the sections were incubated with horseradish peroxidase-conjugated goat anti-rabbit IgG secondary antibody (FLEX/HRP, Dako EnVision) for 30 min at room temperature. The adjacent sections were counterstained with hematoxylin.

### Reagents

Human recombinant TSLP (1398-TS-010), RUXO (TLRL-RUX-3), and ACh (Sigma, A6625-25G) were purchased from R&D Systems, InvivoGen, and Sigma-Aldrich, respectively. All other reagents were purchased from Wako Pure Chemical Industries. The concentration of ACh was determined based on previous experimental studies [5–8]. The concentration of TSLP was selected based on previously reported studies using air–liquid interface cultures of primary human bronchial epithelial cells and cultured dendritic cells [30, 31]. The concentration of RUXO was determined based on previously reported studies on JAK pathway inhibition in cultured porcine airway epithelial cells [32] .

### Statistical analysis

Data are presented as the median and interquartile range [IQR, Q1–Q3]. N denoted the number of pigs. In MCT measurements, beads that moved > 20 μm/sec in the peripheral airway and > 500 μm/sec in the central airway were excluded as an outlier. The beads that stopped moving during the observation period were also excluded. Statistical analyses were performed using JMP Pro 17 and JMP Student ’s Edition 18 (SAS Institute Inc., Cary, NC, USA) and JASP (Version 0.95.2). Statistical differences among groups were evaluated using the Kruskal–Wallis test, followed by Dunn’s post hoc test for multiple comparisons. Statistical significance was set at *p* < 0.05.

## Results

### MCT under physiologically steady-state conditions in both central and peripheral airways

Histograms of the MCT velocities in both airways obtained using the same measurement system are shown in Fig. 1. The median MCT velocity in the central airways was 75.40 μm/sec [IQR: 52.08–114.86], whereas that in the peripheral airways was 2.63 μm/sec [IQR: 2.09–4.35], corresponding to less than one tenth of the central airway velocity.

### Effects of TSLP on MCT velocity in both central and peripheral airways

**Fig. 2:**
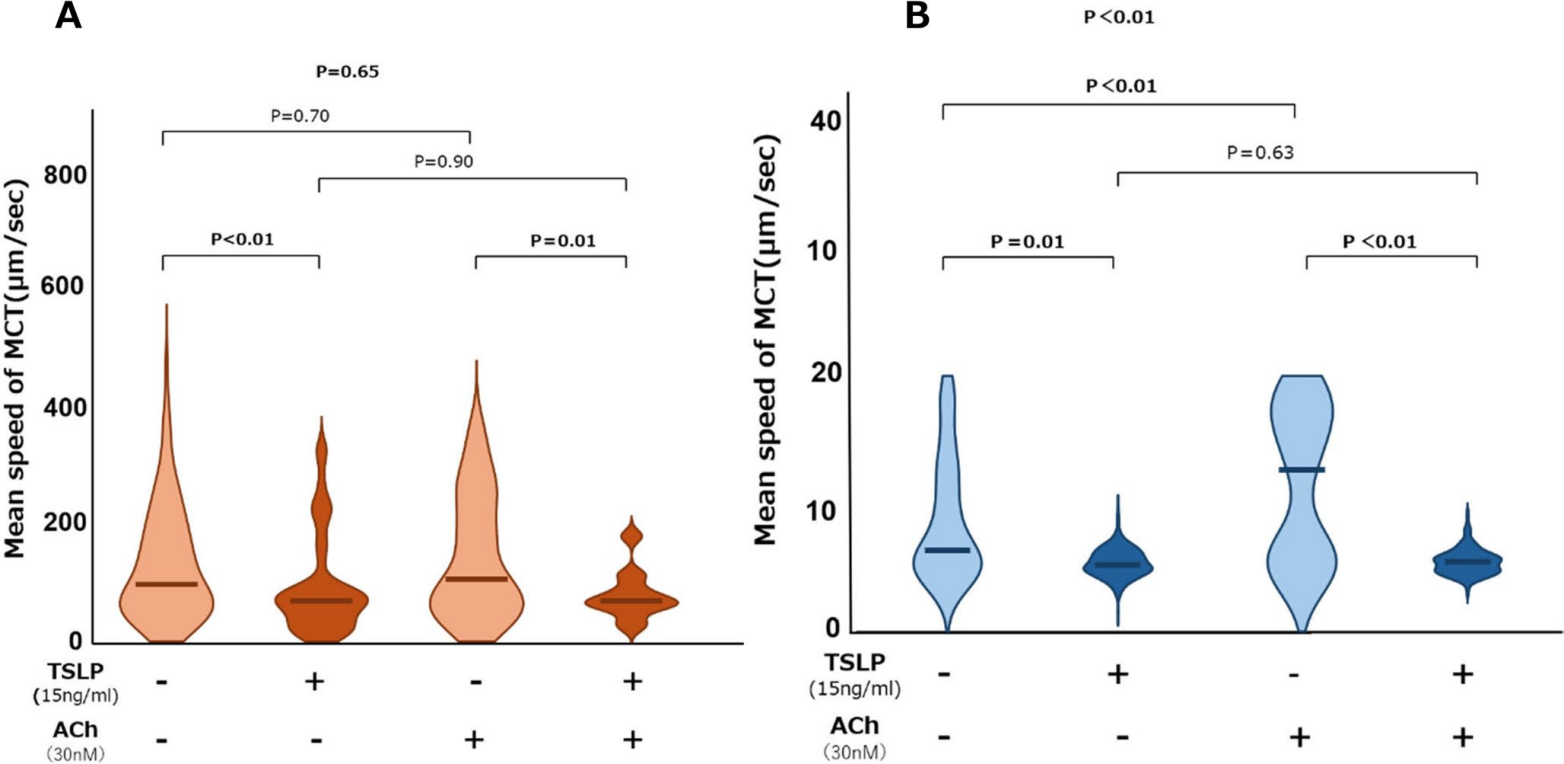
Effects of TSLP on MCT velocity in both central and peripheral airways (*n*=6). The effects of TSLP on the MCT velocity in the central and peripheral airways are shown. Airway tissues are incubated in RPMI medium containing PBS or 15 ng/mL TSLP for 24 h. In the central airways, physiological concentrations of ACh do not alter MCT velocity, whereas TSLP significantly reduces the MCT velocity regardless of ACh stimulation. In the peripheral airways, ACh significantly enhances MCT velocity, and TSLP significantly decreases MCT velocity irrespective of ACh stimulation

The addition of TSLP produced a significant effect both in central and peripheral airways (*p* < 0.01). In the central airways, MCT velocity was consistently significantly decreased by TSLP in both the absence (without TSLP: 99.11 μm/sec.  [60.34–188.20] vs. with TSLP: 69.80 μm/sec. [34.05–94.36], *p* = 0.02) and presence of ACh stimulation (without TSLP: 102.40 μm/sec.  [61.14–228.40] vs. with TSLP: 72.81 μm/sec.  [61.36–101.50], *p* = 0.01) (Fig. 2). In the peripheral airways, TSLP similarly reduced MCT velocity irrespective of ACh stimulation (without ACh: 6.33 μm/sec.  [5.08–12.20 μm/sec] vs. 5.21 μm/sec.  [4.61–6.08 μm/sec], *p* < 0.01; with ACh: 13.21 μm/sec.  [5.77–17.45 μm/sec] vs. 5.46 μm/sec.  [4.74–5.96 μm/sec], *p* < 0.01). These results demonstrate that TSLP decreases MCT velocity in both the central and peripheral airways, regardless of physiologically low concentrations of ACh. In contrast, ACh itself did not significantly affect MCT velocity in the central airways (99.11 μm/sec.  [60.34–188.20 μm/sec] vs. 102.40 μm/sec.  [61.14–228.40 μm/sec], *p* = 0.70), whereas it significantly enhanced MCT velocity in the peripheral airways (6.33 μm/sec.  [5.08–12.21 μm/sec] vs. 13.21 μm/sec.  [5.78–17.45 μm/sec], *p* = 0.01). Although the addition of physiologically low concentrations of ACh was intended to reproduce in vivo conditions and enable prolonged observation (several tens of minutes), it unexpectedly enhanced MCT and may have influenced the results through increased mucin secretion. Therefore, subsequent evaluations of the peripheral airway MCT were performed without ACh stimulation using the measurements obtained at 4 min. Collectively, these findings suggest that TSLP partially inhibits MCT velocity in both the central and peripheral airways, with an approximately 30% reduction in the central airways and a 20–60% reduction in the peripheral airways.

### Effects of TSLP on CBF in both central and peripheral airways

**Fig. 3:**
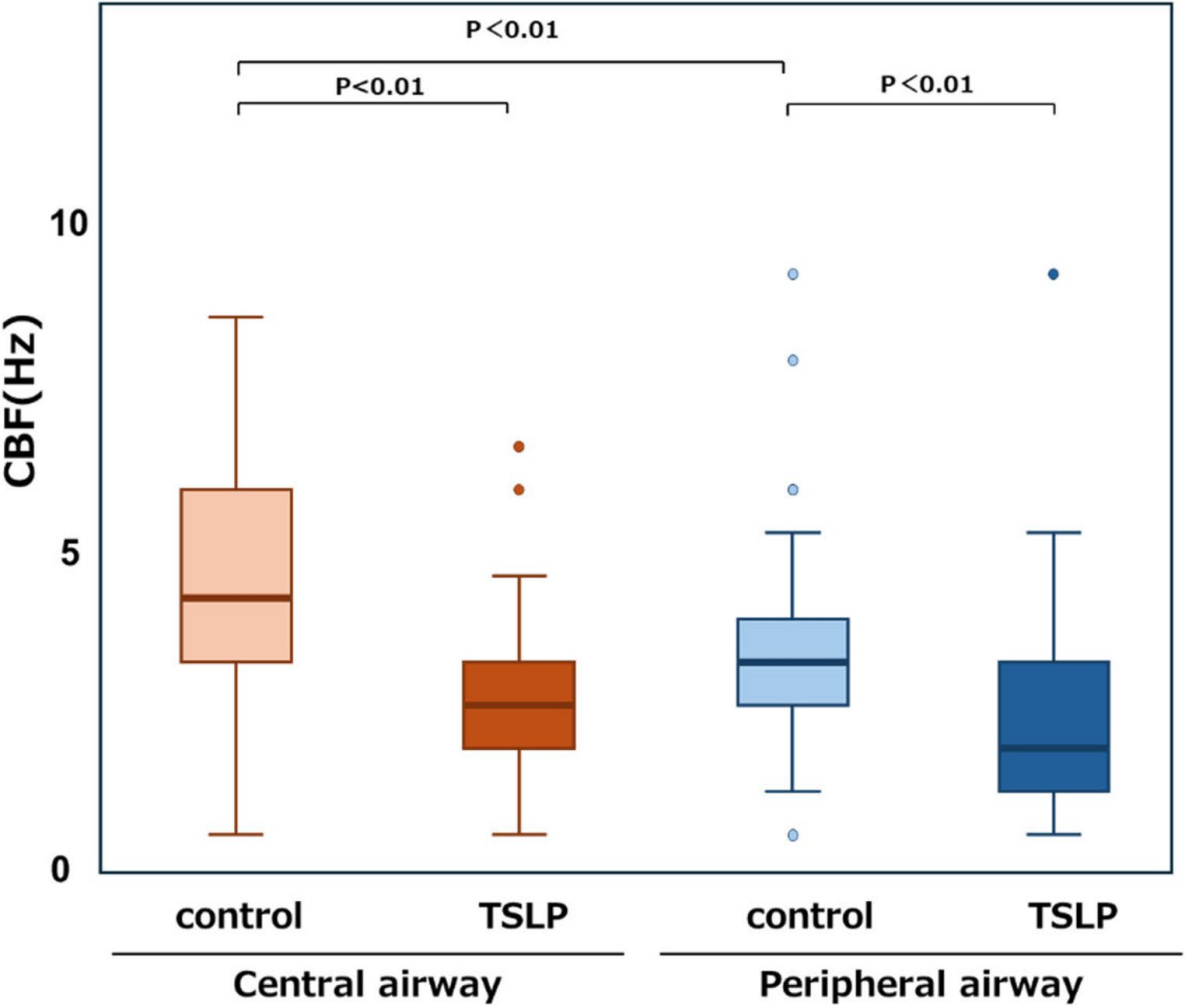
Effects of TSLP on CBF in both central and peripheral airways (*n*=5). The effects of TSLP on CBF are shown. CBF is measured using an inverted microscope equipped with a high-speed camera. Airway tissues are incubated in RPMI medium containing PBS or 15 ng/mL TSLP for 24 h. In both central and peripheral airways, TSLP significantly reduces CBF

MCT velocity is determined by three major factors: (i) the synthesis and secretion of mucins and other mucus proteins, (ii) hydration of the airway surface, and (iii) ciliary beating [33]. Among these, ciliary beating primarily depends on two parameters: CBF and stroke pattern [34]. Given that mucociliary clearance is mainly driven by CBF [35], stroke variation is considered to exert a small effect on the regulation of MCT velocity. Therefore, we examined the effect of TSLP on CBF. To avoid the confounding effects of ACh observed in MCT experiments, CBF was measured without ACh stimulation. A significant difference was observed in CBF (*p* < 0.01). Under basal conditions, the CBF of ciliary cells derived from peripheral airways was significantly lower than that of ciliary cells derived from central airways (4.33 Hz [3.33–6.00 Hz] vs. 3.33 Hz [2.67–4.00 Hz], *p* < 0.01; Fig. 3). Furthermore, TSLP significantly reduced the CBF of ciliary cells in both central (4.33 Hz [3.33–6.00 Hz] vs. 2.66 Hz [2.00–3.33 Hz], *p* < 0.01) and peripheral (3.33 Hz [2.67–4.00] vs. 1.33 Hz [2.00–3.33], *p* < 0.01) airways (Fig. 3). These findings suggest that TSLP partially reduces the CBF in both the central and peripheral airways, with approximately 40% reduction in the central airways and 60% reduction in the peripheral airways.

### TSLPR expression in both central and peripheral airways

**Fig. 4:**
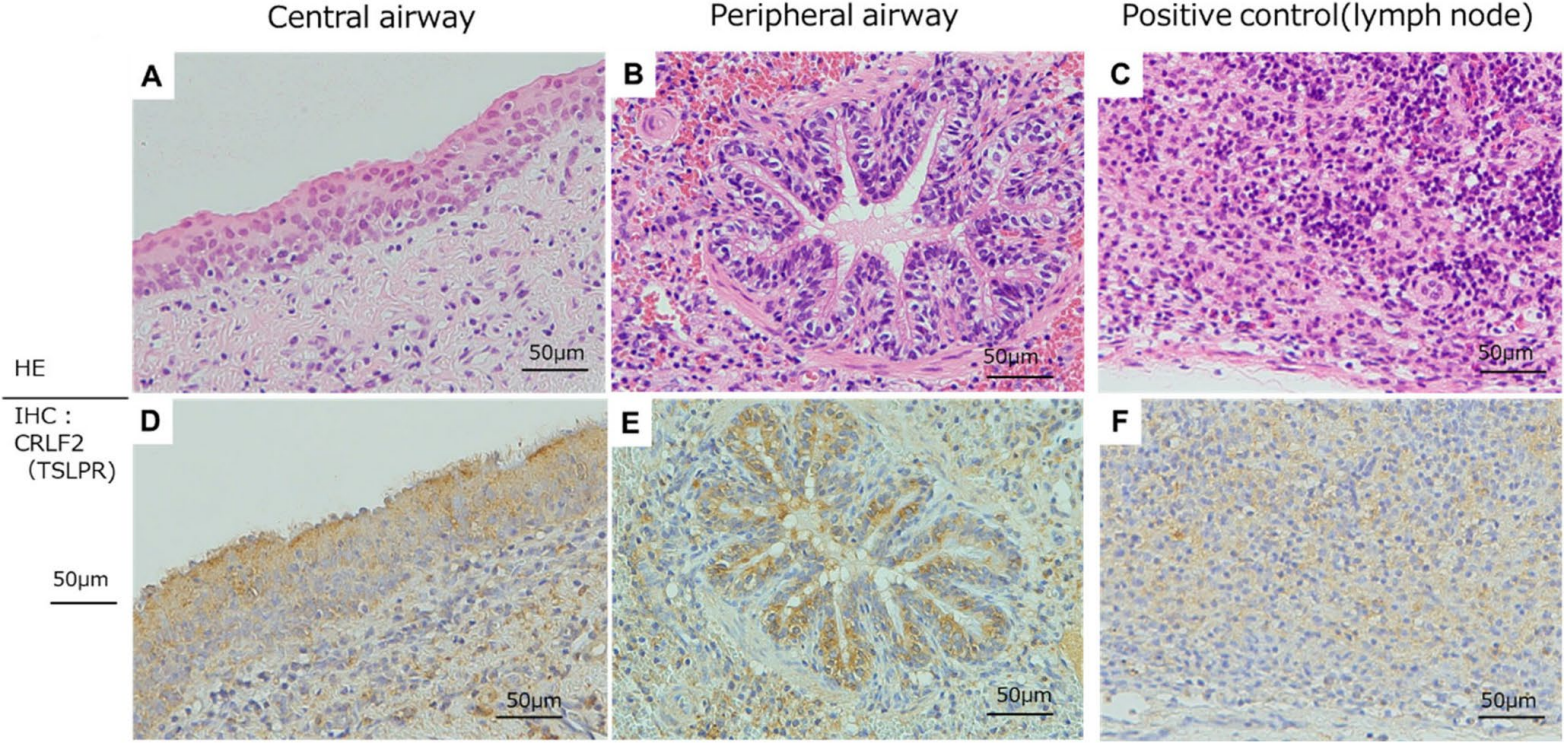
TSLP receptor expression of in central and small airways. Representative images of immunohistochemical staining for the TSLP receptors are shown. **A–C** Hematoxylin–eosin staining. **D–F** Immunostaining with an anti-TSLP receptor antibody. Positive TSLP receptor immunoreactivity is detected in the ciliated epithelium of the central airways (**D**), peripheral airways (**E**), and lymph nodes (**F**)

The results of immunohistochemical staining of TSLPR expression in swine airway tissues are shown in Fig. 4. TSLPR was positively expressed in ciliated epithelial cells in both the central and peripheral airways, confirming TSLPR expression in swine airway tissues.

### Involvement of the JAK signaling pathway in TSLP-induced reduction of MCT velocity

**Fig. 5:**
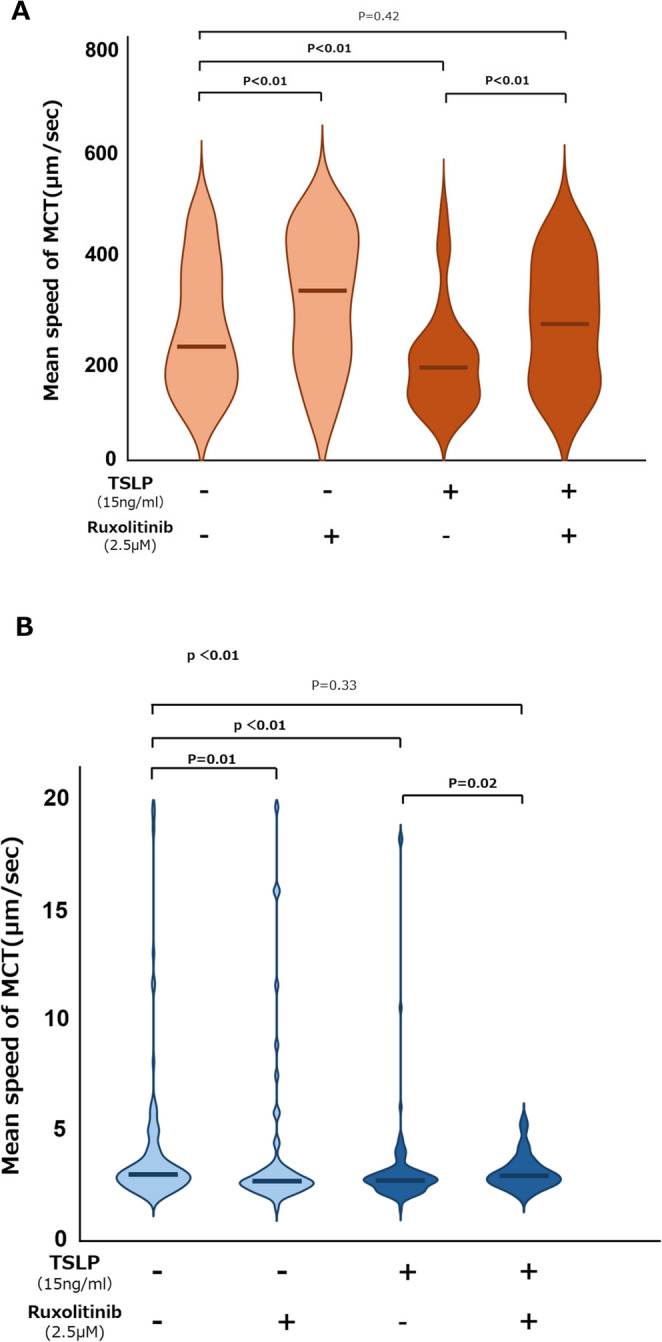
Involvement of the JAK signaling pathway in TSLP-induced reduction of MCT velocity (*n*=5). The effects of the JAK inhibitor RUXO on TSLP-induced suppression of MCT velocity are shown. Airway tissues are incubated in RPMI medium containing PBS or 15 ng/mL TSLP ± 2.5 μM RUXO for 24 h. In the central airways (**A**), MCT velocity is measured after stimulation with 30 nM ACh, RUXO significantly inhibits the TSLP-induced decrease in MCT velocity. In the peripheral airways, MCT velocity is measured without ACh stimulation, and RUXO significantly blocks the TSLP-induced reduction in MCT velocity (**B**)

**Fig. 6:**
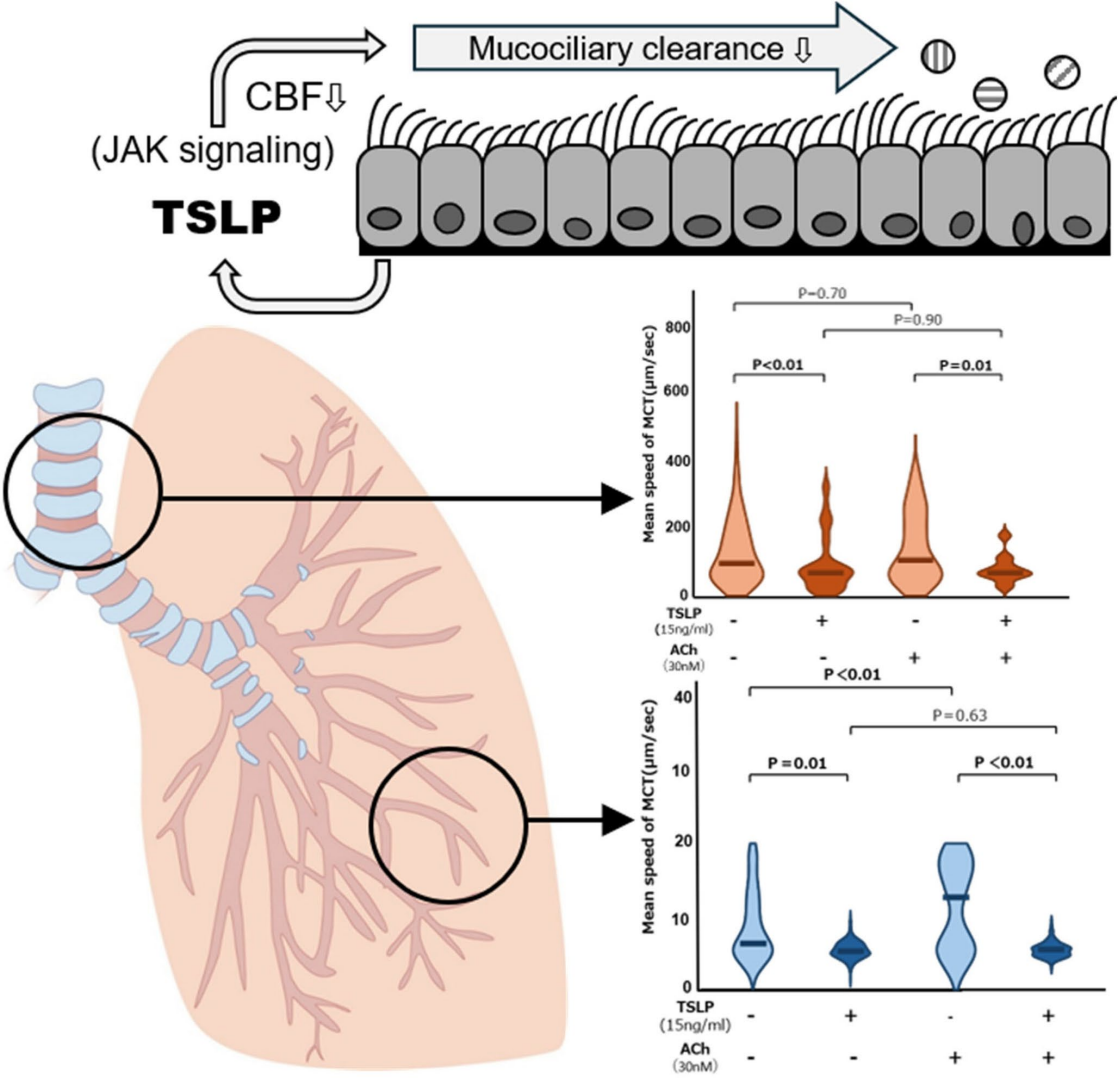
Graphical abstract. In this study, central and peripheral airways were isolated and used for experimentation from freshly obtained porcine airways immediately after slaughter. TSLP addition partially inhibited MCT velocity in both the central and peripheral airways. Administration of TSLP reduced CBF via JAK signaling, resulting in a decrease in mucociliary transport velocity

TSLP is known to bind to its receptor complex, consisting of TSLPR and interleukin (IL)-7 receptor α, and activate cytokine production via the JAK1/2 signaling pathway. Thus, we used the JAK inhibitor RUXO to investigate whether the TSLP-induced reduction in MCT velocity occurred via the TSLPR–JAK signaling pathway [36]. A statistically significant difference was observed in the central airways (*p* < 0.01), and in the peripheral airways (*p* < 0.01). Under stimulation with physiologically low concentrations of ACh (30 nM), RUXO abolished the TSLP-induced decrease in MCT velocity in the central airways (TSLP: 182.6 μm/Sect. [124.2–228.0 μm/sec] vs. TSLP/RUXO: 270.8 μm/Sect. [161.9–378.7 μm/sec], *p* < 0.01). The MCT velocity in the TSLP/RUXO group did not significantly differ from that in the control group without TSLP and RUXO (270.8 μm/Sect. [161.9–378.7 μm/sec] vs. 222.4 μm/Sect. [157.2–255.5 μm/sec], *p* = 0.42; Fig. 5A). In the peripheral airways, where ACh stimulation was omitted to avoid its confounding effects, RUXO similarly abolished the TSLP-induced reduction in MCT velocity (TSLP: 2.78 μm/Sect. [2.44–3.12 μm/sec] vs. TSLP/RUXO: 2.97 μm/Sect. [2.63–3.46 μm/sec], *p* = 0.03), and there was no significant difference when compared with the control group (control: 3.04 μm/Sect. [2.59–3.70 μm/sec] vs. TSLP/RUXO: 2.97 μm/Sect. [2.63–3.46 μm/sec], *p* = 0.33; Fig. 5A, Fig. 6B). These results suggest that TSLP-induced reduction in MCT velocity is mediated by the TSLPR–JAK signaling pathway.

## Discussion

The current study examined differences in MCT velocity and the responses to TSLP stimulation using porcine airways. We initially found that MCT velocity differed between the central and peripheral airways without any stimulation and that physiologically low ACh concentrations increased MCT velocity only in the peripheral airways. Thus, we subsequently investigated the effects of TSLP on MCT velocity. The results showed that TSLP reduced MCT velocity in both the central and peripheral airways (Fig. 2), partly due to a decrease in CBF (Fig. 3). Furthermore, TSLPRs were expressed in ciliated airway epithelial cells (Fig. 4), and TSLP inhibition of MCT velocity was completely abolished by pre-incubation with a JAK inhibitor (Fig. 5). To the best of our knowledge, no previous study has directly compared MCT velocities in the central and peripheral airways using the same measurement method. This study is the first to report that the inhibitory effects of TSLP on MCT may be mediated by the TSLPR–JAK signaling pathway and that MCT characteristics differ between the central and peripheral airways (Fig. 6). Collectively, the findings support that TSLP-induced MCT impairment in peripheral airways may contribute to mucus plug formation in bronchial asthma.

Mucus plugs originate from peripheral airways [23, 37]. Importantly, mucus plug formation plays a crucial role in severe treatment-resistant asthma, correlating with airflow limitation and poor disease control [38, 39]. Eosinophil activation by IL-5 and mucin synthesis induced by IL-13 contribute to mucus plug formation, and TSLP likely plays a pivotal upstream role by amplifying these signaling pathways. An effective MCT can prevent mucus plug accumulation, but MCT dysfunction has been reported in asthma [40, 41]. Although the underlying mechanisms have not been fully elucidated, the present findings help clarify the potential mechanisms involved in mucus plug formation in asthma. Our results are consistent with those of recent studies showing that tezepelumab not only improves asthma control and lung function, but also reduces mucus plugging [42].

Our findings suggested that TSLP reduced MCT velocity by activating the TSLPR–JAK pathway (Fig. 5). TSLPR expression was confirmed in swine bronchial tissues (Fig. 4). TSLPR and IL-7Rα protein expressions in human bronchial epithelial cells have also been previously reported [43]. However, although involvement of the JAK pathway has been indicated, the downstream signaling responsible for ciliary motion impairment remains unclear. TSLP activates the nuclear factor kappa B /p38 mitogen-activated protein kinase pathway through the TSLPR–JAK axis [44]. This in turn enhances the expressions of cyclooxygenase-2 and inducible nitric oxide synthase, leading to excessive production of prostaglandins, nitric oxide, and reactive oxygen species. These mediators may suppress CBF and thereby reduce MCT [45, 46]. Additionally, TSLP upregulates tumor growth factor (TGF)-β expression in dendritic and smooth muscle cells [47, 48]. TGF-β has been reported to suppress ciliary motion via SMAD signaling and oxidative stress pathways [49, 50]. Although our study did not address chronic exposure, long-term TSLP stimulation under inflammatory conditions may activate the PI3K/AKT and mechanistic target of rapamycin pathways. This can lead to repression of cilia-related genes and impaired ciliary motion [51, 52]. Thus, TSLP impairs the MCT through both short- and long-term mechanisms.

Under steady-state conditions, MCT velocity was significantly different between the peripheral and central airways (Fig. 1), consistent with findings of previous studies using cultured airway epithelial cells [13]. This difference may be due not only to the CBF variation observed in our study, but also to previously reported peripheral airway features, including shortened cilia and reduced numbers of ciliated cells [13]. In addition, regional differences in epithelial electrolyte and fluid transport may affect ASL volume and mucus hydration, while differences in mucin biology may influence mucus viscoelasticity. These factors may collectively contribute to the reduced basal MCT velocity observed in the peripheral airways. In the current study, ACh increased MCT velocity only in the peripheral airways (Fig. 2). This preferential response may reflect regional differences in cholinergic regulation along the airway tree. The lower basal MCT velocity in the peripheral airways may allow a greater detectable response to ACh stimulation. In addition, differences in muscarinic receptor distribution, ACh-mediated airway constriction, and epithelial ion/fluid transport may modulate airway surface liquid dynamics and thereby influence MCT velocity [53]. These findings suggest that parasympathetic regulation of mucociliary transport may differ between central and peripheral airways. Given that long-acting muscarinic antagonists have shown beneficial effects on the small airways in asthma and chronic obstructive pulmonary disease [54, 55], our results underscore the importance of site-specific pharmacological interventions tailored to distinct airway regions.

Our findings demonstrated a significant reduction in basal MCT velocity in the peripheral airways, probably because of known physiological gradients in electrolyte transport and mucin composition in these regions. Regarding electrolyte transport, the hydration of the ASL, which is crucial for efficient ciliary beating, exhibits a distinct regional gradient. Previous studies have indicated that peripheral airways possess a higher capacity for Na+ absorption via epithelial sodium channels (ENaC) and a relatively lower capacity for Cl- secretion compared to proximal airways [56]. This shift toward net fluid absorption in the periphery likely results in a thinner ASL layer, thereby increasing friction between the mucus layer and the ciliary tips, which subsequently suppresses the basal MCT velocity. Furthermore, regional differences in mucin biology may also account for the attenuated MCT in peripheral regions. While the proximal airways secrete a mixture of MUC5AC and MUC5B, MUC5B is the predominant gel-forming mucin in the distal and peripheral airways [57, 58]. Given that MUC5B and MUC5AC possess distinct biophysical properties and viscoelasticity, the MUC5B-dominant environment in the narrower lumens of peripheral airways might inherent higher rheological resistance to ciliary transport under basal conditions. Taken together, the reduced basal MCT velocity in peripheral airways observed in this study is likely a physiological consequence of both a thinner ASL layer driven by active fluid absorption and the distinct viscoelastic properties of peripheral mucins.

The current study found that RUXO alone affected the MCT in both the central and peripheral airways (Fig. 5). Although the reason for this remains unclear, the ex vivo experimental system used may have led to a mild activation of the JAK pathway under non-steady-state conditions. However, because the tissues retained responsiveness to TSLP, the basal activation level was not considered excessive, and the reliability of the TSLP stimulation experiments remained intact. Tezepelumab has demonstrated significant clinical benefits in severe asthma, including suppression of type 2 inflammatory biomarkers, reduced exacerbation frequency, and improved lung function (FEV₁) and symptoms [24]. Furthermore, it ameliorates mucus plugs and airway hyperresponsiveness [42].

However, TSLP inhibitors are costly biologics, and their use in Japan is currently indicated only for severe asthma uncontrolled by high-dose inhaled corticosteroids and multiple controller medications, limiting their use to a small patient subgroup. Our finding that TSLP partially inhibits MCT velocity in the peripheral airways, even under non-inflammatory conditions, highlights its potential pathogenic role in earlier disease stages. Given that mucus plugs originate in the small airways and that impaired MCT, reduced antimicrobial activity, and increased mucus viscosity collectively contribute to severe asthma pathophysiology [38, 59–61], early intervention targeting TSLP signaling may be beneficial. As inhaled drugs have limited small airway penetration, the development of affordable, orally available agents that selectively modulate TSLP–JAK signaling in the peripheral airways represents a promising therapeutic strategy.

A key strength of this study is its ex vivo approach, which allowed for the evaluation of MCT differences in a physiologically relevant environment. However, the study also has some limitations. We did not perform detailed analyses of protein or gene expression in ciliated airway epithelial cells. Knockdown or over-expression experiments to elucidate the underlying molecular mechanisms were also not conducted. Moreover, although the ex vivo model closely reflected in vivo physiology, potential influences from constituent cells other than ciliated cells could not be ruled out. While immune cell involvement is unlikely under these conditions, secondary responses mediated by structural cells (e.g., dendritic cells) may exert an effect. Another limitation is the use of healthy swine airways, which may not fully replicate the human asthmatic airway environment. In addition to potential species differences between swine and humans, the present experiments evaluated the acute effects of TSLP exposure in otherwise healthy airways. In contrast, mucus plugging in asthma typically develops in the setting of chronic airway inflammation, epithelial remodeling, goblet cell hyperplasia, altered mucin production, and impaired mucus clearance. Furthermore, the present experimental system was not designed as an asthma model and therefore did not reproduce the complex inflammatory milieu of established asthma, including sustained type 2 inflammation, inflammatory cell infiltration, and airway remodeling. Accordingly, although our findings suggest that TSLP can acutely impair MCT, caution is required when extrapolating these results to mucus plugging in established asthma. It is also possible that the mechanisms underlying TSLP responsiveness differ between human and porcine airways. The available porcine TSLP sequence shares approximately 64% amino acid identity with the corresponding C-terminal region of human TSLP. In addition, recombinant human TSLP has been reported to induce biological responses in porcine experimental systems, supporting the biological relevance of using human TSLP in the present porcine airway model [62]. In addition, airway remodeling associated with asthma, including changes in the goblet cell population, may affect these responses. Consequently, chronic responses that were not evaluated in the present study may also play a role [63]. To address these limitations, we are developing an experimental system using primary human airway epithelial cells.

## Conclusion

TSLP plays a crucial role in the disruption of airway defense mechanisms through MCT impairment in peripheral airways. This suggests that the TSLP–JAK signaling pathway may represent a potential therapeutic target for mucus plug formation, a key pathological feature in refractory asthma. Furthermore, there are physiological differences between the central and peripheral airways, highlighting the necessity for developing therapeutic strategies that consider the distinct pathophysiological characteristics of each airway region. We hope that our findings demonstrating the importance of JAK signaling in peripheral airway MCT regulation will help push for further drug development in this area.

## Data Availability

No datasets were generated or analysed during the current study.
